# Endoscopic approach and stereotactic radiosurgery for a posterior third ventricular Central Neurocytoma – case report and literature review

**DOI:** 10.1016/j.ijscr.2020.02.042

**Published:** 2020-02-25

**Authors:** Jolyn Khoo, Gert Tollesson

**Affiliations:** Neurosurgery, Greenslopes Private Hospital, Newdegate Street, Greenslopes, QLD 4120, Australia

**Keywords:** CN, Central Neurocytoma, CSF, cerebrospinal fluid, EMA, epithelial membrane antigen, ETV, endoscopic third ventriculostomy, GFAP, glial fibrillary acid protein, GTR, gross total resection, H&E, haematoxylin and eosin, MRI, magnetic resonance imaging, STR, subtotal resection, Case report, Central Neurocytoma, Third ventricle, Endoscopic, Radiosurgery

## Abstract

**Introduction:**

Central Neurocytomas (CN) are a rare intracranial tumour, most often arising in the lateral ventricles and presenting with obstructive hydrocephalus. Isolated lesions in the third ventricle are uncommon. We present the fourth reported case of posterior third ventricular CN successfully managed surgically via endoscopy, allowing for concurrent biopsy and therapeutic endoscopic third ventriculostomy (ETV). Stereotactic radiosurgery was administered for the residual lesion. A brief review of CNs and previous similar cases is also provided.

**Presentation of case:**

A 58-year-old male presented with progressive decline in cognition and gait. Subsequent Magnetic Resonance Imaging revealed obstructive hydrocephalus secondary to a posterior third ventricular lesion. An endoscopic biopsy and concurrent cerebrospinal fluid diversion by ETV was performed. Pathological analysis was consistent with a CN with positivity to Synaptophysin. MIB-1 proliferation index was 1%. There was good clinical recovery, and the patient underwent adjuvant stereotactic radiosurgery 1.5 months post-surgery.

**Discussion:**

Due to the rarity of CNs arising from the third ventricle, there are only three previous reports of these approached endoscopically. Such a technique allows for good visualisation of the lesion, and therapeutic ETV to relieve obstructive hydrocephalus. This case supports this approach as a valid, minimally invasive option. Additionally, this is the first case to report the MIB-1 proliferation index, contributing to future outcome evaluation of endoscopic approaches to typical (MIB-1 < = 2%) verses atypical (MIB-1 > 2%) CNs.

**Conclusion:**

Endoscopic biopsy with concurrent ETV and adjuvant stereotactic radiosurgery is a valid treatment option for deep seated isolated small posterior third ventricular CNs.

## Highlights

• Central Neurocytomas are a rare intracranial tumour, often presenting with obstructive hydrocephalus.
• Isolated lesions in the posterior third ventricle are an uncommon location for Central Neurocytomas.
• An endoscopic approach to these allows for concurrent biopsy and therapeutic cerebrospinal fluid diversion.
• Adjuvant Stereotactic Radiosurgery improves outcomes of survival and local control in subtotal resection.
• Together, this is a viable treatment for deep-seated, posterior third ventricular Central Neurocytomas.

## 1 Introduction

Central Neurocytomas (CN) are a rare form of intracranial neoplasm, accounting for 0.1–0.5% of cases with peak incidence between 20–40 years of age. Lesions are typically located in the anterior half of the lateral ventricles, with attachment to the septum pellucidum [1,2]. CNs arising from the third or fourth ventricles are even more uncommon [3]. Due to compounded rarity of isolated third ventricular CNs, there is limited literature guiding management of these. In line with SCARE guidelines [4], we present a case of a posterior third ventricular CN with MIB-1 proliferation index 1% that was surgically biopsied via an endoscopic approach with concurrent endoscopic third ventriculostomy (ETV), and adjuvant radiosurgery for residual disease. The endoscopic technique has only been used in three other previous cases of posterior third ventricular CNs [[5], [6], [7]], but none with documented proliferation indexes. We review the previous similar cases, and current literature regarding the management of CNs. We also briefly review two previous reports of CNs located just adjacent to the third ventricle that were also managed endoscopically [8].

## 2 Presentation of case

A 58-year-old male presented to the emergency department with progressive decline in cognition and gait with recurrent falls over days, on a background of generalised lethargy for 3–4 months. There was a sight headache but no nausea. Past medical history was significant only for asthma, with no regular medications. Clinically the patient had a Glasgow Coma Score of 15, though there was a slowness of thinking below baseline. He had a wide based unsteady gait, but no other focal neurology.

**Fig. 1:**
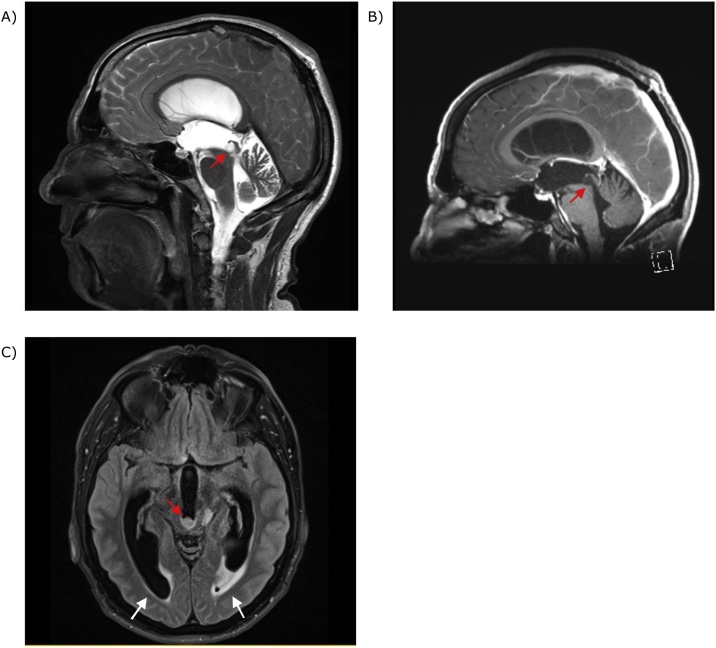
MRI brain showing: A - Sagittal T2 sequence with posterior third ventricular lesion obstructing aqueduct (arrow); B – Same lesion (arrow) appearing hypointense and non-enhancing on sagittal T1 with contrast sequence; C – Axial FLAIR sequence with lesion appearing hyperintense (red arrow). Obstructive hydrocephalus with prominent temporal horns and transependymal flow (white arrows).

**Fig. 2:**
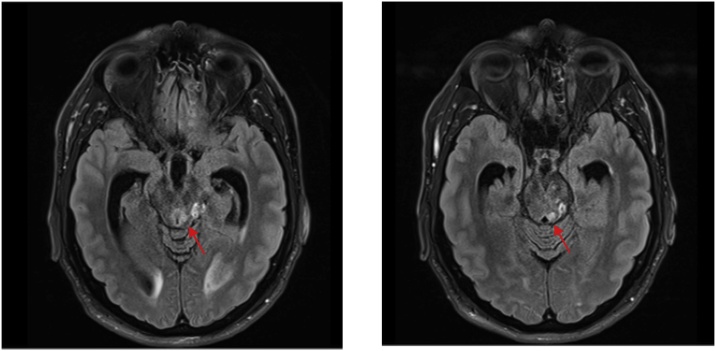
MRI brain axial FLAIR sequences showing tumour extension along left periaqueduct (arrows).

A Computed Tomography scan of the brain was significant for triventriculomegaly with no obvious lesion. Subsequent Magnetic Resonance Imaging (MRI) brain revealed a 7 mm round lesion in the posterior third ventricle obstructing the aqueduct. It was hyperintense on T2 and FLAIR, and hypointense on T1 with no contrast enhancement (Fig. 1). The lesion extended along the left periaqueduct to the superior fourth ventricle (Fig. 2). Obstructive hydrocephalus with transependymal flow was also demonstrated (Fig. 1).

Given the deep-seated location and the resultant obstructive hydrocephalus, an endoscopic approach was planned to allow for both an ETV and endoscopic biopsy. Under navigation guidance, a standard ETV was performed via a right frontal burr hole with balloon fenestration. A second, more anterior right burr hole was required to achieve the trajectory needed to reach the lesion. Intraoperatively, the lesion appeared as a pinkish, friable mass just anterior to the opening of the cerebral aqueduct. Biopsy samples were obtained using rongers. As the lesion extended periaqueductally towards the fourth ventricle, gross total resection (GTR) could not be attempted safely.

Post operatively, the patient noticed a marked improvement in cognition and gait. A day one brain MRI with cerebrospinal fluid (CSF) flow studies confirmed a patent ETV, with a reduction in the degree of hydrocephalus. The lesion itself appeared similar.

**Fig. 3:**
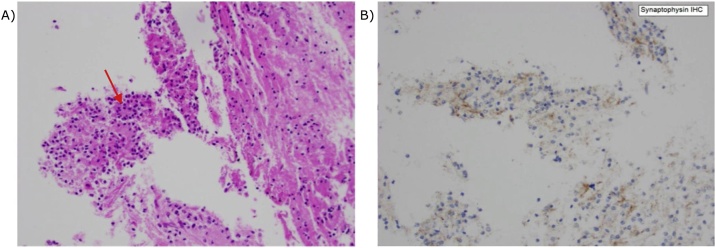
Histopathological slides of tumour: A – standard H&E stain showing increased cellularity with rosetting (arrow); B – positive immunohistochemistry staining for Synaptophysin.

Histological analysis of the tumour showed mild increase in cellularity with rosetting, but no high-grade features on standard Haematoxylin and Eosin (H&E) stain. Immunohistochemistry was moderately positive for Synaptophysin (Fig. 3) and positive for Oligo 2, with variable staining for Glial fibrillary acid protein (GFAP). Epithelial membrane antigen (EMA) was negative and there is diffuse positivity for S-100. Both IDH 1R132H and P53 were negative. The MIB-1 index was 1%. Fluorescence in situ Hybridization was negative for both 1P 19Q co-deletion and Epidermal Growth Factor Receptor amplification. These findings were most in keeping with a diagnosis of CN.

**Fig. 4:**
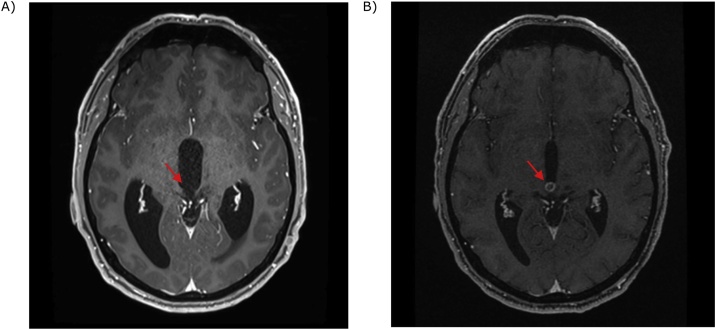
MRI brain showing: A – Pre-operative Axial T1 sequence with contrast, lesion is non-enhancing (arrow) and hydrocephalus is evident; B – same sequence at 5 months post-operation demonstrating rim enhancing lesion (arrow) and resolution of hydrocephalus.

A 1 month MRI brain and spine showed ongoing ETV patency with a further reduction in hydrocephalus. The lesion was unchanged, and there was no spinal metastasis. The patient underwent stereotactic radiosurgery with 29 Gy in 5# to the residual lesion 1.5 months after surgery. At 5 months post operation, MRI brain demonstrated a decrease in size of the lesion with new rim enhancement reflecting treatment effect. The ETV remained patent and there was resolution of hydrocephalus (Fig. 4). The patient returned to work uneventfully and remained clinically well at 5 months post operation.

## 3 Discussion

CNs were first proposed as a distinct tumour type by Hassoun et al. in 1982 [9], and are considered a benign neoplasm with a grade two classification by the World Health Organisation [1,2]. CNs are very rare, making up only 0.1–0.5% of central nervous system tumours, and peak incidence is between 20–40 years of age [1,2]. Lesions typically arise from the lateral ventricles, and may extend into the distal ventricles. Isolated origin from the third or fourth ventricle is less common [3]. Clinically, patients may present with obstructive hydrocephalus, most frequently from blockage of the Foramen of Monro for typically located tumours [1,2].

Histopathologically, CNs can resemble oligodendrogliomas or ependymomas under light microscope with standard stains, making diagnosis complex. Immunochistochemistry is generally required to guide diagnosis, though it is recognised that markers can have cross-positivity with other tumours and more research is required to develop diagnostic accuracy [1,2]. Positivity for synaptopsin is considered the main marker for CNs, and negative testing for EMA, vimentin and neuron specific enolase support CN over oligodendrogliomas and ependymomas. GFAP is usually absent in CNs, however cases of positive results have been reported before suggesting a precursor cell with differential bipotential towards both glial and neuronal [1,2]. Neuron-specific nuclear protein is more likely to be positive in CNs than oligodendrogliomas [2,10], while the converse is found for Olig2 [1,10]. The MIB-1 proliferation index is considered an important prognostic indicator in CNs where patients with an index >2% have a higher chance of recurrence and lower survival at 10 years. It has been proposed that CNs with an index >2% be considered ‘atypical’ and more aggressive, and have been found to metastasize along the neural axis [1,11,12].

The mainstay management of CNs involves GTR. An analysis of 438 patients by Rades and Schild [13] found that for both typical and atypical lesions, patients with GTR had significant improvement in survival and local control when compared to subtotal resection (STR) alone. Adjuvant radiotherapy is advocated for residual lesions in STR, and significantly bettered outcomes of survival and local control. The use of adjuvant stereotactic radiosurgery for STR has also been reported, and may potentially be more effective than fractionated radiotherapy with a trend towards better survival and local control rates, though this did not reach statistical significance [14].

Lesions of the posterior third ventricle are deep and challenging to reach surgically. Small lesions are amendable to an endoscopic approach which allows for minimal access surgery, good visualisation of the lesion, and ability to perform concurrent CSF diversion via an ETV. Due to the rarity of CNs, and its unlikeliness to arise from the third ventricle, there are only three previous reports where endoscopic technique was utilised for management of isolated third ventricular CNs (Table 1) [[5], [6], [7]]. Of note, an additional two cases of CNs located just adjacent to the posterior third ventricle have likewise been successfully managed with this approach [8].

**Table 1:** Summary Endoscopic Management of Isolated Third Ventricular CNs.

Article	Age/ Gender	MRI	Surgery	Adjuvant Treatment	Outcome
Javedan et al. [5]	54/M	• Enhancing lesion, size unknown • Obstructive hydrocephalus	• Endoscopic biopsy • Friable, well defined, pink grey tumor • ETV via seperate burr hole	• Stereotactic radiosurgery 2 weeks post surgery (18 Gy at 50% isodose line)	• Symptom improvement and return to work • Patent ETV and resolution of hydrocephalus • Minimal tumour decrease on 25 month MRI
Park et al. [6]	79/F	• 18 mm multilobulated strongly enhancing lesion • obstructive hydrocephalus	• ETV • Endoscopic biopsy of pink, friable tumour with mildly increased vascularity	• Stereotactic radiosurgery 1 week post surgery (14 Gy with 50% isodose lines)	• Symptom improvement of gait and cognition • Reduction of tumour from 1.8 cm to 1.4 cm at 3 month MRI
Romano et al. [7]	37/F	• 18 mm moderately enhancing lesion • Obstructive hydrocephalus	• Endoscopic GTR with aid of diode laser and ronger • ETV	• Nil	• Symptom improvement, resolution of diplopia • No recurrance up to 36-month MRI
Khoo et al. (present paper)	59/M	• 7 mm non enhancing lesion with extension along aqueduct • obstructive hydrocephalus	• ETV • Endoscopic biopsy	• Stereotactic radiosurgery 1.5 months post surgery (29 Gy in 5#)	• Symptom improvement especially gait and cognition • Reduction in lesion size at 5 month MRI

Javedan et al. [5] were the first to introduce this technique in a 54-year-old patient with obstructive hydrocephalus from a posterior third ventricular CN. Endoscopic biopsy of the lesion was undertaken but adherence to the surrounding tissue and vessels prevented more extensive debulking. An ETV was also performed, and the patient underwent adjuvant stereotactic radiosurgery of the residual tumour two weeks later. There was good clinical recovery, and a 25-month follow-up MRI showed minimal decrease of the lesion, a patent ETV, and resolution of the hydrocephalus. The authors did not mention the MIB-1 proliferation index.

Park et al. [6] describe on a 79-year-old female with a multilobulated 1.8 cm posterior third ventricular CN also presenting with obstructive hydrocephalus. Similarly, an ETV was performed along with endoscopic biopsy of the lesion. Adjuvant stereotactic radiosurgery was undertaken at one week post-surgery, and a 3-month progress MRI show a decrease in the lesion size to 1.4 cm with the patient remaining well at 8 months. It was suggested that stereotactic radiosurgery could be a suitable treatment alternative to deeply seated CNs in older patients. Unfortunately, the MIB-1 proliferation index was also not reported.

In the report by Romano et al. [7] a 37-year-old female presented with obstructive hydrocephalus and left abducent nerve palsy secondary to a posterior third ventricular CN. With the aid of a diode laser and rongeur via an endoscopic technique, GTR of the lesion was achieved and an ETV was also performed. The patient did well postoperatively with resolution of the diplopia. There was no adjuvant radiotherapy or radiosurgery, and subsequent post-operative MRIs up to 36 months did not show any residual or regrowth of the CN. This was the only reported case where GTR was attained, with the use of a diode laser instrumental to achieving this safely. The MIB-1 proliferation index was not mentioned.

A side noteworthy paper to mention is by Gomes et al. [8] where two cases of CNs located just adjacent to the third ventricle were similarly managed endoscopically. A 58-year-old male with a pineal region lesion, and a 21-year-old female with an aqueductal region lesion both presented with symptoms of obstructive hydrocephalus, and underwent endoscopic biopsy along with ETV. Post-operative resolution of the hydrocephalus and good clinical outcomes were achieved, and no adjuvant radiotherapy or radiosurgery was given. Neither case had the MIB-1 proliferation index reported. The authors emphasized that CNs in the pineal and aqueductal regions appear similar to tectal gliomas, and can be successfully approached endoscopically.

This current case report represents the fourth reported successful usage of endoscopic approach for a small posterior third ventricular CN and CSF diversion by an ETV. Because the lesion extended down the left periaqueduct to the superior fourth ventricle, GTR was not achievable. The patient underwent adjuvant radiosurgery of the residual lesion as recommended in previous reviews of this rare tumour. Of the four reports, this is the only case to have a documented MIB-1 proliferation index, which will contribute to future evaluation of endoscopic approaches for typical versus atypical posterior third ventricular CNs. Consideration of use of a diode laser to aid endoscopic resection was felt as an important learning point from the review of previous cases, though this is operator dependent and requires technical training.

## Sources of funding

None.

## Ethical approval

This study is exempt from ethical approval in our institution.

## Consent

Written informed consent was obtained from the patient for publication of this case report and accompanying images. A copy of the written consent is available for review by the Editor-in-Chief of this journal on request.

## Author contribution

Jolyn Khoo – Primary author.

Gert Tollesson – Reviewer.

## Registration of research studies

N/A.

## Guarantor

Jolyn Khoo.

## Provenance and peer review

Not commissioned, externally peer-reviewed.

## Declaration of Competing Interest

None.
